# Attenuated Replication-Competent Herpes Simplex Virus Expressing an ECM-Modifying Transgene Hyaluronan Synthase 2 of Naked Mole Rat in Oncolytic Gene Therapy

**DOI:** 10.3390/microorganisms11112657

**Published:** 2023-10-29

**Authors:** Jussi Palomäki, Kiira Kalke, Julius Orpana, Liisa Lund, Fanny Frejborg, Henrik Paavilainen, Hannu Järveläinen, Veijo Hukkanen

**Affiliations:** 1 Institute of Biomedicine, University of Turku, Kiinamyllynkatu 10, 20520 Turku, Finland; jussi.palomaki@utu.fi (J.P.);; 2 Pharmaceutical Sciences Laboratory, Faculty of Science and Engineering, Åbo Akademi University, 20520 Turku, Finland; 3 Department of Internal Medicine, Satakunta Hospital District, Satasairaala Central Hospital, Sairaalantie 3, 28500 Pori, Finland

**Keywords:** herpes simplex, cancer, oncolytic virus, extracellular matrix, hyaluronan synthase, naked mole rat

## Abstract

Herpes simplex virus (HSV) has proven successful in treating human cancer. Since the approval of talimogene laherparepvec (T-VEC) in 2015, HSV has been thoroughly researched to discover novel mechanisms to combat cancer and treat other diseases. Another HSV-based drug, beremagene geperpavec (B-VEC), received approval in 2023 to treat the rare genetic disease dystrophic epidermolysis bullosa, and was also the first clinically approved HSV vector carrying an extracellular matrix (ECM)-modifying transgene. The ECM is a network of macromolecules surrounding cells, which provides support and regulates cell growth and differentiation, the disruption of which is common in cancer. The naked mole rat (NMR) has a thick ECM and a unique mutation in the *hyaluronan synthase 2* (*HAS2*) gene, which has been linked to the high cancer resistance of the species. To study the effect of this mutation in human cancer, we have developed an attenuated, replication-competent HSV vector expressing the *NMR-HAS2* gene. The viral replication, transgene expression and cytotoxic effect of the novel vector was studied in glioma cells. Our results show that an attenuated, replication-competent HSV vector expressing a foreign ECM-modifying transgene, namely *HAS2*, provides an effective tool to study and combat cancer in humans.

## 1. Introduction

### 1.1. Oncolytic HSV

Herpes simplex virus (HSV) has multiple advantages as a gene therapy vector. The large genome of the HSV consists of both essential and non-essential genes, of which more than 30 kbp may be deleted and replaced with foreign DNA without losing the replication competence of the virus [1,2]. The viral genome, residing in the viral capsid as linear double-stranded DNA, is released during the infection into the cell nucleus, where it circularizes and remains in an extrachromosomal state without being integrated into the host’s DNA [3]. This minimizes the risk for adverse effects, such as the insertional oncogenesis reported with retroviral vectors [4]. The cytolytic effect of the wild-type HSV is relatively high, which is desirable in the case of cancer cells but may cause risks such as inflammation in clinical settings. To decrease these risks, vector safety and cell survival can be enhanced by removing certain viral genes, such as the ones encoding the neurovirulence factor infected cell protein 34.5 (ICP34.5) or regulatory factors, such as ICP4, ICP22 and ICP27 [5]. The neurovirulence factor ICP34.5 is of special interest in gene therapy in the central nervous system (CNS), as it dephosphorylates and thus activates eukaryote initiation factor 2 (eIF2), which is essential for HSV replication in healthy neurons, but not in tumor cells, enabling the selective targeting of tumors in the CNS [6,7,8,9,10]. Most of the HSV vectors currently studied have the gene for ICP34.5 deleted [11].

The aim of oncolytic gene therapy is to induce lysis in cancer cells while causing minimal harm to healthy cells [12]. The first oncolytic virus approved for clinical use by the European Medicines Agency (EMA) and the Food and Drug Administration (FDA) was talimogene laherparepvec (T-VEC), an HSV-1 vector with the viral gene coding for ICP34.5 deleted and replaced by a gene coding for granulocyte-macrophage colony-stimulating factor (GM-CSF), a cytokine that enhances immune responses against cancer cells [13,14,15]. T-VEC was the first virus approved for clinical use against melanoma, and since its approval, numerous clinical trials have been initiated to test its effectiveness against other types of skin cancers and solid tumors [16]. Another type of oncolytic HSV currently in clinical trials is HF10, a spontaneously mutated HSV-1 strain with deletions and the overexpression of certain viral genes. HF10 shows enhanced tumor selectivity, viral replication and oncolytic potential, although its mechanism is not completely clear and is under active investigation, parallel with clinical studies [17,18]. In a preclinical study, several other circulating wild-type HSV strains with promising oncolytic potential were identified, and of these, a cell-associated phenotype and rapid replication tend to correlate with a high oncolytic potential [11].

The use of HSV as a therapeutic vector is not limited to its use as an oncolytic vector. The HSV genome has the capability of the high-level expression of large transgenes, providing a venue to study the effects of different kinds of foreign genes and gene products targeting specific cancer characteristics, such as the tumor microenvironment. Targeting the extracellular matrix (ECM) with HSV-derived therapeutics has already proven successful, as phase III clinical trials for B-VEC, a replication-defective HSV-1 expressing gene coding for collagen type VII, have been completed [19]. The FDA has recently approved this vector as a treatment for a rare genetic disease, dystrophic epidermolysis bullosa [20].

### 1.2. Cancer and the ECM

The ECM is a network of macromolecules that surrounds the cells, provides support for tissues and regulates cell growth and differentiation. The ECM is in a constant state of remodeling, regulated by bidirectional interactions between the ECM components and the cells in it. Cell–ECM contact and signaling molecules stored in the matrix modulate cell proliferation and migration. Cells also secrete factors that build, modify and cleave the ECM [21,22]. Common structural macromolecules of the ECM include fibronectin, which forms the initial scaffolding for other ECM components in matrix remodeling [23,24], collagen, which acts as the main load-bearer of the ECM [25,26], and hyaluronan, which fills the remaining space by binding water [27]. The remodeling of the ECM is a tightly regulated process, and its disruption leads to scar hypertrophy [28] and is also a common feature of cancer [29]. 

### 1.3. Hyaluronan

Hyaluronan (HA) is an anionic, linear polysaccharide produced on the cell membrane by hyaluronan synthases (HAS) [27]. Cells interact with HA via the cell adhesion molecule CD44 [30] and use it as a scaffold for migration. HA acts as a modulator of inflammation and cell proliferation. Its effect is highly dependent on the cellular context, and its molecular size [31]. 

HA oligomers act as ECM-damage-associated molecular patterns (ECM-DAMPs), which activate pro-inflammatory cytokine release, the proliferation of endothelial cells and resident fibroblasts in the matrix and the production of ECM precursors, with the aim to repair the damaged ECM [22]. The stiffening of the ECM leads to the activation of transforming growth factor β (TGF-β) and other signaling pathways inside the cells, leading to cell differentiation, cell cycle arrest, replicative senescence and senescence-associated secretory phenotype (SASP), which further amplifies the secretion of ECM component precursors and pro-inflammatory cytokines [32,33,34]. As the physiological damage repair cascade progresses, the pro-inflammatory cytokine release stimulated by ECM-DAMPs attracts CD4+ T cells to clear the senescent cells, leading to the return of normal tissue function. If the process is disrupted by the building up of ECM-DAMPs or apoptosis-resistant senescent cells, the tissue stays in the state of chronic inflammation, leading to DNA damage and cancer progression [35,36].

Low-molecular-weight HA (LMW-HA) activates pro-inflammatory cytokine release through toll-like receptor 4 (TLR4) [37], which promotes cell proliferation [38]. Furthermore, LMW-HA is pro-angiogenic, as it has been shown to enhance the expression of vascular endothelial growth factor (VEGF) A and VEGF receptor 1 (VEGFR1), e.g., in liver endothelial cells [39]. 

The effects of high-molecular-weight HA (HMW-HA) are generally regarded as anti-inflammatory, anti-angiogenic and anti-tumorigenic. Its anti-inflammatory effect has been linked to the downregulation of TLR4-dependent receptor activator of nuclear factor-κB ligand (RANKL) expression [40] and the anti-angiogenic effect of the inhibition of cyclin D1 in vascular smooth muscle cells [41]. In some studies, a high concentration of human HMW-HA has been shown to reduce glioblastoma cell migration [42], but later studies have contradicted this [43]. In a study of human and mouse glioma cell lines, the overexpression of human/mouse *HAS2* had either a tumor-suppressing or -promoting effect, the latter of which occurred when HA-degrading enzymes, such as *hyaluronidase 2* (*HYAL2*), were simultaneously expressed [44]. The overexpression of HA-degrading enzymes has been described in clinical settings as well. For example, the overexpression of *hyaluronidase transmembrane protein 2* (*TMEM2*) is correlated with a significantly lower rate of survival for patients with pancreatic adenocarcinoma [45].

The naked mole rat (NMR) is known for its exceptional longevity and resistance to cancer. Its cancer resistance is linked to a unique mutation in the *HAS2* gene, causing it to produce extremely high-molecular-weight HA (EHMW-HA) that is over five-fold heavier than human or mouse HA. EHMW-HA induces the p16^INK4a^-pathway in NMR cells, leading to cell cycle arrest [46]. EHMW-HA has also been shown to induce apoptosis in human and mouse breast cancer cells through the induction of p53 protein expression and to inhibit tumor xenograft growth in nude mice [47]. Transgenic C57BL/6 mice expressing the *NMR-HAS2* gene show decreased spontaneous cancer incidence, increased resistance to chemically induced tumorigenesis, an improved health span, a 4.4% increase in their median lifespan and a 12.2% increase in their maximum lifespan [48].

In this study, we utilized HSV to ramp up EHMW-HA production by expressing the *NMR-HAS2* gene, known to produce EHMW-HA, with the aim of studying the effects of deliberately changing the HA balance in glioma cells and their surroundings, as well as the effects of a foreign transgene on the oncolytic potential of the HSV vector itself.

## 2. Materials and Methods

### 2.1. Cell Lines

Vero cells (CCL-81, ATCC, Manassas, VA, USA) were maintained in Dulbecco’s Modified Eagle Medium (DMEM) (Lonza, Basel, Switzerland), 7% heat-inactivated fetal calf serum (FCS) (Gibco, Carlsbad, CA, USA) and gentamycin (Gibco). For plaque titration assay, human immunoglobulin G (IgG) (Kiovig, Takeda, Wien, Austria) was added to the growth medium. U373MG cells, currently reclassified as U251 (HTB-17, ATCC) but here referred to with their original name for continuity with earlier publications from our group, were maintained in DMEM with 5% heat-inactivated FCS, 1% GlutaMAX (Gibco) and gentamycin.

### 2.2. Viruses

**Figure 1:**
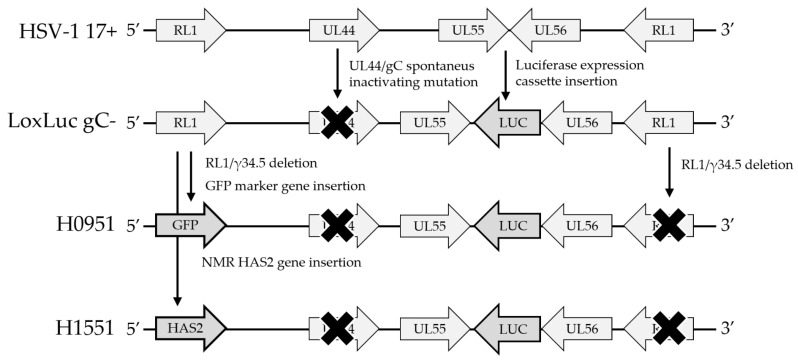
Development pathway of *γ_1_34.5*- and *gC*-negative HSV vectors H0951 and H1551. The light gray arrows represent HSV genes, the dark gray arrows represent the transgenes and the crosses represent gene deletions.

To study the effect of NMR EHMW-HA on human cancer, we developed an HSV vector to express the *NMR-HAS2* gene (XM_004838010.2). An HSV recombinant strain derived from HSV-1 17+, with a deletion in the open reading frame of the *RL1/γ_1_34.5* gene (ICP34.5) and *luciferase* expression cassette insertion between open reading frames *UL55* and *UL56* (HSV-1(17+)Lox-Luc-Δγ_1_34.5-Zeo) [49], was used as a backbone. *NMR-HAS2* cDNA was ordered from Life Technologies/Thermo Fisher (Waltham, MA, USA). Plasmid work was performed in the *E. coli* strain DH10β. *NMR-HAS2* cDNA was cloned under murine cytomegalovirus (CMV) immediate early promoter into the *RL1/γ_1_34.5* gene locus of HSV’s bacterial artificial chromosome (BAC). The HSV strain (17+)-based BAC was kindly supplied by Prof. Beate Sodeik, MHH Hannover, Germany. Successful cloning was verified with restriction fragment analysis (Supplementary Figure S1). Modified HSV-BAC was transfected with MBS Mammalian Transfection kit (Agilent Technologies, Santa Clara, CA, USA) into Vero cells to produce the vector, which was designated as H1551. A similar method was used earlier by our group to develop *γ_1_34.5*-deleted HSV vectors H1052 and H0951 expressing a *green fluorescent protein* (*GFP*) marker gene [50]. The HSV BAC used to develop H1551 and H0951 harbored an inactivating frameshift mutation in the *UL44* gene that codes for glycoprotein C (Table 1, Figure 1). A stock of the transgenic virus vector (H1551) was prepared from infected cells and stored in 9% milk (Valio, Finland). An HSV vector with a *GFP* marker as a transgene (H0951) and vector backbone without a transgene in *γ_1_34.5* gene locus (HSV-1(17+)Lox-Luc) [49] (hereon: LoxLuc gC-) were used as controls.

### 2.3. Virus Infections

Virus replication was studied in U373MG cells. Cells on 24-well plates were infected with 1 pfu/cell of the H1551 vector (*NMR-HAS2*), and H0951 (*GFP*) was used as a control. Supernatant samples were collected at the time points 6 h post infection (hpi), 12 hpi, 18 hpi, 24 hpi, 30 hpi and 48 hpi for viral release assay, and the cells were lysed with TRI reagent (Thermo Fisher Scientific, Waltham, MA, USA) for gene expression assay.

### 2.4. Viral Release Assay

Viral release was measured by plaque titration assay. Supernatants from different time points were collected and dilutions 1:10^2^–1:10^7^ were assayed in 24-well plates containing Vero cells, with human immunoglobulin G (IgG) added to the growth medium to prevent viral spread through the medium. Infections were terminated with methanol at the time point 96 hpi and stained with crystal violet, and the plaques were counted.

### 2.5. Gene Expression Assay

Gene expression was measured via reverse-transcription polymerase chain reaction (RT-qPCR) as described earlier [51]. TRI reagent-lysed cell samples from different time points were collected, mRNA was isolated as instructed by the manufacturer, treated with DNase (Thermo Fisher, cat. EN0521, Waltham, MA, USA) and reverse transcribed into complementary DNA (cDNA) with RevertAid H Reverse Transcriptase (Thermo Fisher, cat. EP0441) and random hexamer primers (Thermo Fisher, cat. SO142) as described earlier [51,52]. The amounts of cDNA were quantified with primers (Table 2) mixed with SYBR green enzyme (Thermo Fisher, cat. K0253) using QIAGEN (Venio, The Netherlands) Rotor-Gene Q (2-Plex) with Rotor-Gene Q Software 2.3.1.49. Primers designed for the *NMR-HAS2* gene were used to quantify *NMR-HAS2* gene expression. Copy numbers of HSV *virus protein 16* (VP16) gene mRNA were measured as a marker for viral replication. Additionally, the mRNA copy numbers of *protein kinase R* (*PKR*) gene, *interleukin 29* (*IL-29*) gene and *interferon β* (*IFNβ*) gene were measured as markers of cellular response to the virus. The RT-qPCR results were normalized to the copy numbers of the housekeeping gene *glyceraldehyde 3-phosphate dehydrogenase* (*GAPDH*) in each sample. The sequences of the primers are shown in Table 2.

### 2.6. Oncolytic Assay

The oncolytic potential of the vector was measured by CellTiter-Glo^®^ cell viability assay (Promega, Madison, WI, USA). A series of 0.001–1 pfu/cell infections were established in U373MG cells in 96-well plates, and the cell viability assay was performed according to the manufacturer’s instruction at the time points 24 hpi and 48 hpi. Additionally, to compare the oncolytic potential of the vector to that of the parental strain and a series of wild-type HSVs, 2 pfu/cell infections were established in U373MG cells in 96-well plates, and the cell viability assay was performed at the time point 96 hpi, in parallel with the study on the wild-type HSV strains, the method of which was described earlier [11].

### 2.7. Statistical Analysis

Statistical analyses were performed with Analysis ToolPak in Microsoft^®^ Excel^®^ for Microsoft 365 MSO (version 2306 Build 16.0.16529.20164). Statistical significances were calculated with one-way ANOVA by comparing two individual groups at a time, with the threshold of significance set as a *p*-value of <0.05.

## 3. Results

**Figure 2:**
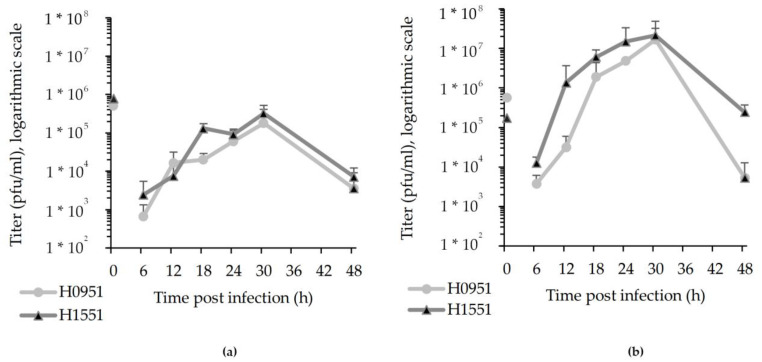
The replication patterns of H1551 (*NMR-HAS2*) and H0951 (*GFP*) in U373MG (**a**) and Vero (**b**) cells, based on extracellularly released progeny virus from 1 pfu/cell infection on a logarithmic scale. The closed circles and triangles in the graph represent the titer of the released virus. The capped bars represent the standard error of the mean (SEM) (n = 3). Time point of 0 hpi in the graph represents the titer of the medium used to infect the cells.

The viral release of the H1551 vector (*NMR-HAS2*) and the control vector H0951 (*GFP*) from U373MG glioma cells was measured via the plaque titration of supernatant samples in Vero cells, and a growth curve was produced (Figure 2a). An additional growth curve was produced from a 1 pfu/cell infection in Vero cells, which, in contrast to U373MG cells, are non-malignant cells (Figure 2b). In U373MG cells, a slight fluctuation in the viral release of H1551 was noticeable, and at the time point 12 hpi, it temporarily slows down to below that of the control vector. In Vero cells, this fluctuation was not noticeable.

**Figure 3:**
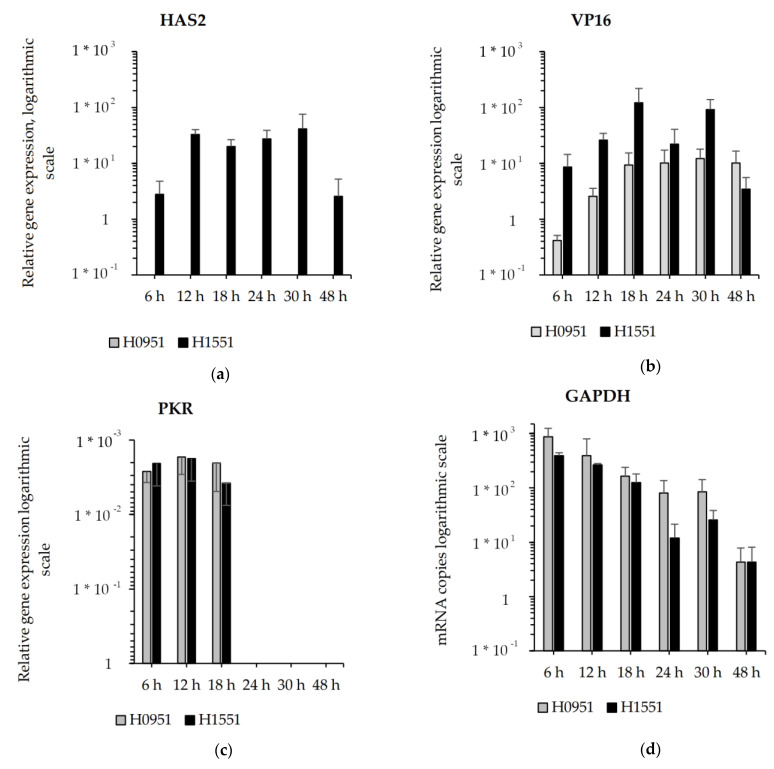
mRNA expression in glioma cell line U373MG infected with H1551 (*NMR-HAS2*) and H0951 (*GFP*). The expression patterns of *NMR-HAS2* (**a**), *VP16* (**b**) and *PKR* (**c**), normalized to housekeeping gene *GAPDH* (**d**), are shown on a logarithmic scale. The columns represent the copy numbers of the mRNA, and the capped bars represent the SEM (n = 3).

To measure the viral gene and transgene expression, an RT-qPCR assay was conducted on the mRNA samples collected from the infected cells. The *NMR-HAS2* RT-qPCR assay shows an effective transgene expression peak at 12 hpi from H1551, while no *NMR-HAS2* mRNA expression is detectable from the control vector (Figure 3a). *VP16* was chosen as a marker for the viral gene expression, and its expression in H1551 is in line with the viral release pattern at the time points 18–30 hpi, as a slight fluctuation in the same direction is noticeable in the gene expression. Up until 30 hpi, the viral gene expression is noticeably higher compared to that of the control vector (Figure 3b). 

At the early stage of infection, a minor *PKR* signal was detected, a sign of cells trying to shut down the viral replication (Figure 3c). The vectors did not show an *IL-29* or *IFNβ* response in U373MG cells 

The gene expression of the housekeeping gene *GAPDH* decreased during the infection. At the time points 6 hpi, 12 hpi and 24 hpi, the *GAPDH* expression was measurably lower in the cells infected with H1551 compared to that of the control, suggesting the increased cytotoxicity of the *NMR-HAS2*-expressing vector in U373MG cells (Figure 3d).

**Figure 4:**
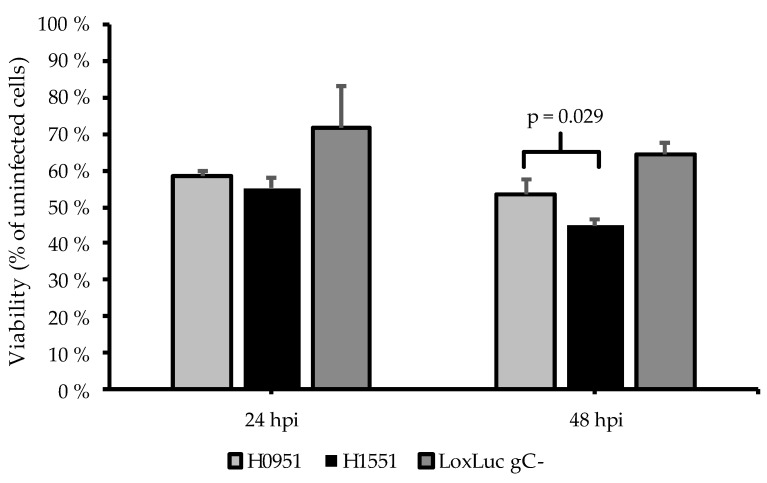
Cytotoxicity in glioma cell line U373MG. The cells were infected with 1 pfu/cell of H1551 (*NMR-HAS2*), H0951 (*GFP*) and LoxLuc gC- (no transgene in the *γ_1_34.5* locus) and measured for cell viability 24 hpi and 48 hpi, utilizing a luminescent assay. The viability was determined by comparing the signal derived from the infected cells to that derived from the non-infected cells. The columns represent the percentage of viability of the cells, and the capped bars represent the SEM (n = 3). H1551 (*NMR-HAS2*) showed higher cytotoxicity compared to that of the controls at both time points, and at the time point 48 hpi, the difference was statistically significant (*p* = 0.029).

To analyze the possible enhanced oncolytic effect of H1551, a cell viability assay was conducted with U373MG cells on a 96-well plate at the time points 24 hpi and 48 hpi in an infection series ranging from 0.001 to 1 pfu/cell infection. H0951 was used as a control and the backbone virus LoxLuc gC- (no transgene in the *γ_1_34.5* gene locus) was used as an additional control. In both time points, H1551 showed an increased cytotoxicity compared to that of the controls in the 1 pfu/cell infection (Figure 4).

**Figure 5:**
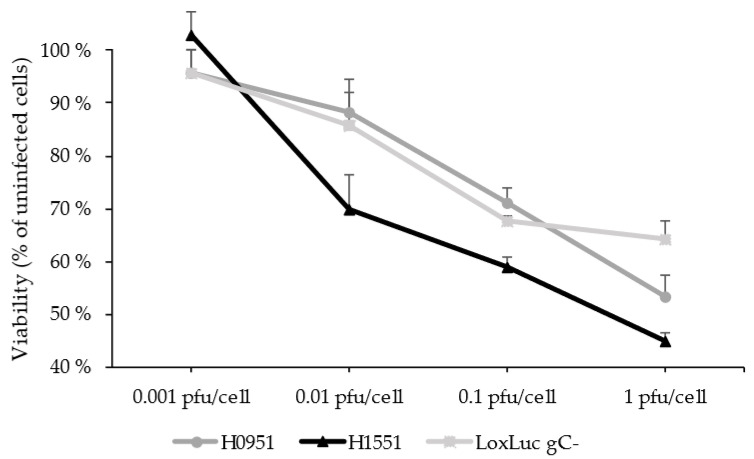
Cytotoxicity in glioma cell line U373MG at 48 hpi. The cells were infected with 0.001–1 pfu/cell of H1551 (*NMR-HAS2*), H0951 (*GFP*) and LoxLuc gC- (no transgene in the *γ_1_34.5* locus) and measured for cell viability at 48 hpi, utilizing a luminescent assay. The viability was determined by comparing the signal derived from the infected cells to that from the non-infected cells. The closed circles represent the percentage of viability of the cells, and the capped bars represent the SEM (n = 3).

Lower pfu/cell infections did not show significant cytotoxicity at 24 hpi, but at 48 hpi, the cytotoxicity was noticeable starting from the 0.01 pfu/cell infection, and H1551 showed a higher cytotoxicity compared to that of the controls (Figure 5).

**Figure 6:**
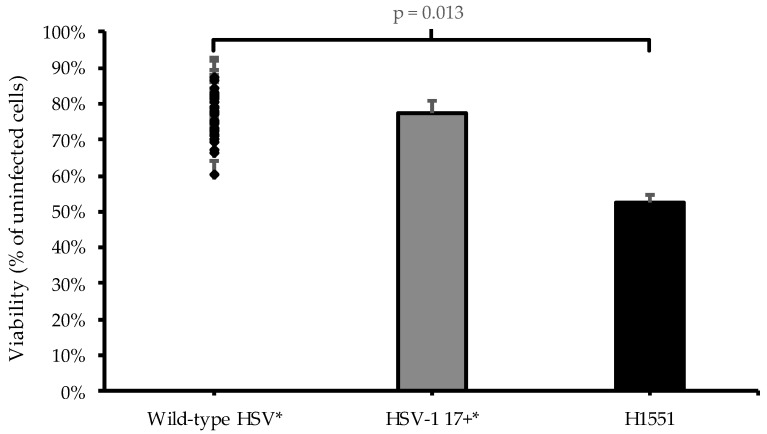
Cytotoxicity in glioma cell line U373MG. The cells were infected with 2 pfu/cell of H1551 (*NMR-HAS2*), in parallel with a study on the oncolytic potential of wild-type HSV strains, and measured for cell viability at 96 hpi, utilizing a luminescent assay. The viability was determined by comparing the signal derived from the infected cells to that derived from the non-infected cells. The columns and the closed diamonds represent the percentage of viability of the cells, and the capped bars represent the SEM (n = 4). The asterisks represent the data that was published earlier by Kalke et al. [11], the scatter plot on the left represents the cytotoxicity of the studied wild-type HSV strains and the column in the center represents HSV-1 17+, which is the *γ_1_34.5*-positive parental strain from which H1551 is derived. The cytotoxic effect of H1551 was higher compared to each of the published wild-type HSV strains, and the difference was statistically significant (*p* = 0.013).

Finally, an additional cell viability assay was conducted to make a comparison of the oncolytic potential of the H1551 vector and the cytotoxicity data of the parental virus HSV-1 17+ and a series of wild-type HSV strains, published earlier by Kalke et al. [11]. U373MG cells were infected with 2 pfu/cell of H1551, and the cell viability was measured at 96 hpi (Figure 6).

## 4. Discussion

The last decade has uncovered numerous therapeutic genes with the potential to combat cancer, but the pipeline from gene sequencing to clinical treatment is long and laborious. The HSV has been proven both safe and effective in clinic applications as well as a suitable tool for basic and translational research. In this study, we developed an attenuated, replication-competent HSV vector, which expresses the *HAS2* gene of NMR. By doing so, we showed that our HSV construct is capable of effectively expressing foreign genes in human cancer cells.

In both malignant and non-malignant cell lines, the viral release of the vector was uniformly slightly higher than that of the control, suggesting faster viral replication, but the pattern of the viral release did not significantly differ from the control. However, the viral gene expression was significantly higher compared to that of the control, and at the time point 12 hpi, also noticeably higher compared to viral release at the same time point, suggesting a more effective viral replication and more cell-bound phenotype of the *NMR-HAS2*-expressing vector at the early stage of infection (Figure 3b). Based on previous results, these traits tend to correlate with a higher oncolytic potential [11]. The gene expression of the housekeeping gene *GAPDH* decreased faster compared to the control vector, suggesting increased cytotoxicity, which was further confirmed by the cytotoxicity assay, in which the *NMR-HAS2*-expressing vector showed a significantly higher cytotoxicity than that of the controls (Figure 4, Figure 5 and Figure 6). As an unexpected finding, the cytotoxicity of the *NMR-HAS2*-expressing vector was even higher than that of the wild-type HSV strains identified and published earlier [11], which is a promising finding for the *γ_1_34.5*-deleted HSV.

The anti-cancer effect of EHMW-HA in NMR was first hypothesized to be linked to the induction of cell cycle arrest [46] and to a decrease in cell migration [42]. A later study has shown that the transfection of *NMR-HAS2* mRNA can induce apoptosis in human and mouse breast cancer cells in vitro, as well as the inhibition of cancer xenograft growth in nude mice [47]. Our study supports the observation that *NMR-HAS2* may have an anti-cancer effect in humans, and this effect can be induced in glioma cells by expressing the gene with an HSV vector. As the *NMR-HAS2*-expressing vector showed a slightly altered pattern of viral release and viral gene expression, it seems likely that *NMR-HAS2* also affected the behavior of the vector itself. Although further studies, such as in vivo experiments in different cancer models, are needed to identify the exact mechanism of its enhanced oncolytic potential, as well as the precise effect that *NMR-HAS2* has on human cancer cell replication and migration, expressing the gene with an HSV vector provides a straightforward path from laboratory to clinical applications, in the case that further studies also show positive results.

The expression of foreign genes in human cells raises safety and ethical concerns. A patient’s immune system is likely to recognize *NMR-HAS2* as a foreign protein, inducing an immune response against the infected cells. In the context of cancer, this is a desired outcome. The ethical concerns of transgene expression with an HSV vector are minor compared to popular gene editing methods, such as retroviruses and CRISPR, since the HSV DNA carrying the transgene does not integrate into the host’s DNA and therefore does not have the risk of unwanted mutations, especially germline mutations which are currently under major bioethical discussion [57].

## 5. Conclusions

In conclusion, our study shows the expression of a foreign anti-cancer gene as an additional pathway to utilize HSV in cancer research, both in the laboratory setting and in the path to clinical drug development. It also supports the observation that the *NMR-HAS2* gene may have an anti-cancer effect in humans and shows that this effect can be induced with an HSV vector expressing the gene.

## Figures and Tables

**Table 1:** Transgenic HSV vectors derived from HSV-1 17+ strain. All the derivative viruses carry a *luciferase* expression cassette between ORFs *UL55* and *UL56*. H1052 used in our previous studies has the *RL1/γ134.5* gene replaced with a *GFP* marker gene, H0951 has an inactivating mutation in the *UL44* (gC) gene but is otherwise comparable to H1052 and H1551 has the *RL1/γ_1_34.5* gene replaced with the *NMR-HAS2* gene. LoxLuc and LoxLuc gC- do not express transgenes from the *γ_1_34.5* gene locus.

HSV Strain	RL1/γ_1_34.5	UL55/UL56 Insertion	UL44	Notes
17+	+	-	+	Parental strain
LoxLuc	+	Luc	+	[49]
H1052	GFP	Luc	+	[50]
LoxLuc gC-	+	Luc	-	This study
H0951	GFP	Luc	-	This study
H1551	NMR-HAS2	Luc	-	This study

**Table 2:** Primers used in the gene expression assay.

Name	Sequence	References
HAS2 sense	5′-AAGTGGGGTGGAAAACGAG-3′	This study
HAS2 antisense	5′-GTCCAGCATGGTATCGGAGT-3′	This study
VP16 sense	5′-TTTGACCCGCGAGATCCTAT-3′	[50,53]
VP16 antisense	5′-GCTCCGTTGACGAACATGAA-3′	[50,53]
PKR sense	5′-GGCCGCTAAACTTGCATATC-3′	[54]
PKR antisense	5′-GCGAGTGTGCTGGTCACTAA-3′	[54]
IFNβ sense	5′-TCTCCACGACAGCTCTTTCCA-3′	[51,55]
IFNβ antisense	5′-ACACTGACAATTGCTGCTTCTTTG-3′	[51,55]
IL-29/IFNλ1 sense	5′-GGAGCTAGCGAGCTTCAAGA-3′	[51,52]
IL-29/IFNλ1 antisense	5′-GGAAGACAGGAGAGCTGCAA-3′	[51,52]
GAPDH sense	5′-GAGAAGGCTGGGGCTCAT-3′	[51,56]
GAPDH antisense	5′-TGCTGATGATCTTGAGGCTG-3′	[51,56]

## Data Availability

The data presented in this study are available on request from the corresponding author.

## Supplementary Materials

The following supporting information can be downloaded at: https://www.mdpi.com/article/10.3390/microorganisms11112657/s1, Figure S1: H1551 (NMR-HAS2) BAC restriction fragment analysis.
