# Supplementary material for: Attenuated Replication-Competent Herpes Simplex Virus Expressing an ECM-Modifying Transgene Hyaluronan Synthase 2 of Naked Mole Rat in Oncolytic Gene Therapy
Source: Microorganisms. 2023 Oct 29;11(11):2657. doi: 10.3390/microorganisms11112657 (PMC10673056; doi:10.3390/microorganisms11112657)
Supplement: Supplementary file 1 [file microorganisms-11-02657-s001.zip › microorganisms-2665272-supplementary.pdf]

**Supplementary Materials**

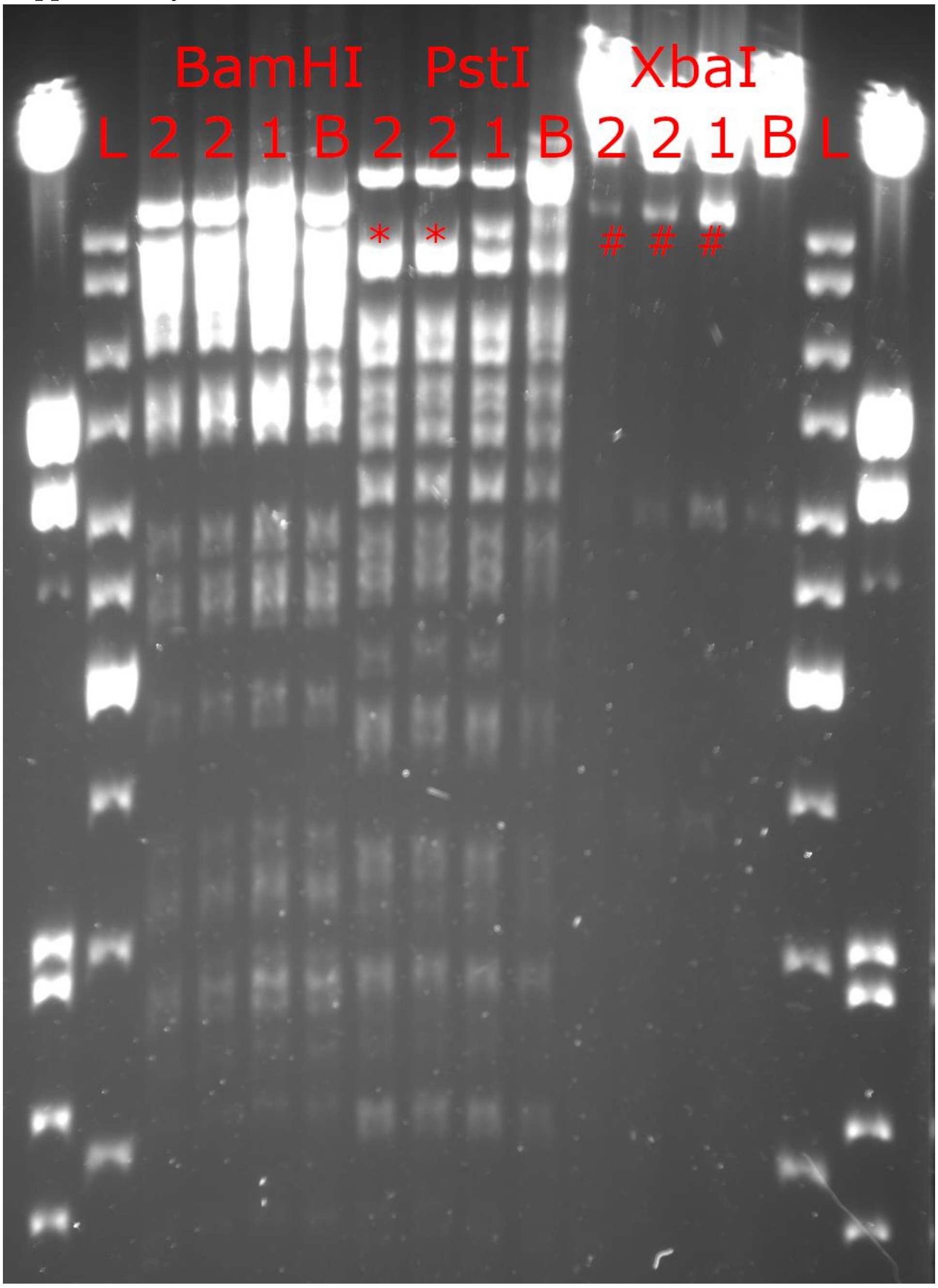


**Supplementary Figure S1**. H1551 (NMR-HAS2) HSV bacterial artificial chromosome (HSV-BAC) restriction fragment analysis. HSV-BAC miniprep was prepared with Macherey-Nagel™ NucleoBond™ BAC 100 Kit (Fisher Scientific, Loughborough, Leicestershire, The United Kingdom) according to manufacturer's instructions. Each HSV-BAC DNA sample (approximately 15 μg) was diluted in 35 μl of solution containing sterile $H_2O$, FastDigest 10X green buffer, and FastDigest BamHI, PstI or XbaI restriction enzyme (Thermo Fisher Scientific, Waltham, MA, USA). Electrophoresis was run in 0.6% Tris- borate-EDTA (TBE)-agarose gels, with GeneRuler mix DNA ladder (Thermo Fisher Scientific), for 24 h at 60 V before imaging. The ladder is represented with a letter L on top of the column. NMR-HAS2 gene has an XbaI restriction site 222 bp downstream of translation initiation site which, compared to unaltered HSV-BAC (represented by letter B on top of the column), produces an additional 11101 bp XbaI-fragment (#) in the restriction pattern of the successful cloning (represented by number 1 on top of the column). After the cloning, 1163 bp of HSV-BAC elements were removed, shortening the 10256 bp PstI-fragment to 9093 bp in the final transfection-ready viral DNA (represented by number 2 on top of the column).
